# Mechanical property changes of glial LC and RGC axons in response to high intraocular pressure

**DOI:** 10.3389/fbioe.2025.1574231

**Published:** 2025-04-28

**Authors:** Bochao Ma, Liu Liu, Yushu Liu, Jifeng Ren, Xiuqing Qian

**Affiliations:** ^1^ School of Biomedical Engineering, Capital Medical University, Beijing, China; ^2^ Department of Medical Engineering, Peking University Third Hospital, Beijing, China; ^3^ School of Special Education and Rehabilitation, Binzhou Medical University, Yantai, Shandong, China; ^4^ Beijing Key Laboratory of Fundamental Research on Biomechanics in Clinical Application, School of Biomedical Engineering, Capital Medical University, Beijing, China

**Keywords:** high intraocular pressure, lamina cribrosa, morphology, mechanical properties, RGC axons

## Abstract

**Introduction:**

Pathological high intraocular pressure (IOP) is an important risk factor for glaucoma. The lamina cribrosa (LC) area in the optic nerve head is the initial site of optic nerve injury for glaucoma. LC deformation caused by elevated IOP will compress the retinal ganglion cells (RGC) axons passing through it, thereby leading to the damage of the RGC axons. The deformation of LC is highly correlated with its mechanical properties. Therefore, changes in mechanical properties of LC with the duration of high IOP is of great significance.

**Methods:**

To investigate the impact of chronic high IOP on the mechanical properties of the LC, rat models were established by cauterizing the superior scleral vein and injecting 5-fluorouracil (5-FU) under the conjunctiva to maintain elevated IOP. The linear elastic properties of the glial LC and RGC axons in affected eyes were measured using atomic force microscopy (AFM) combined with image segmentation techniques. Morphological alterations of the glial LC were assessed using hematoxylin-eosin staining, immunofluorescence staining, and transmission electron microscopy (TEM).

**Results:**

Compared to the control group, the Young's modulus of the glial LC decreased by 35.5%, 74.2%, and 80.6% at 4, 8, and 12 weeks of elevated IOP, respectively. Similarly, the Young's modulus of RGC axons decreased by 45.6%, 70.9%, and 75.9% over the same time points. These findings demonstrate a time-dependent reduction in the mechanical stiffness of both glial LC and RGC axons under chronic high IOP conditions.

**Discussion:**

The progressive decrease in Young's modulus indicated that prolonged high IOP compromises the structural integrity and mechanical properties of the LC and RGC axons. This mechanical weakening likely contributes to the pathophysiological process of optic nerve injury in glaucoma. The present study offers important insights into the biomechanical mechanisms underlying glaucomatous damage, which may guide future research and therapeutic strategies.

## 1 Introduction

Glaucoma is a serious, irreversible blinding eye disease and the second leading cause of blindness in the world (Tham et al., 2014). The pathogenesis of glaucoma is still unclear, but the pathological increase of intraocular pressure (IOP) is an important factor. The lamina cribrosa (LC), a connective tissue structure in the optic nerve head (ONH), supports the retinal ganglion cells (RGC) axons as they pass through the eyeball (Quigley and Addicks, 1980; Midgett et al., 2020). Elevated IOP can lead to LC deformation and further compression of the optic nerve (ON), resulting in the damage of RGC axons (Yan et al., 1994; Midgett et al., 2020; Korneva et al., 2023). The deformation of LC is associated with its mechanical properties (Karimi et al., 2021), and elevated IOP will alter the mechanical properties of LC (Jia et al., 2022). Therefore, studies of mechanical properties change of LC and RGC axons will provide information to analyze the LC deformation more accurately and provide a fundament for the mechanisms of optic nerve damage in glaucoma.

Previous research has shown that optic nerve damage occurs initially in the LC area and is related to the LC morphology of glaucoma patients (Ivers et al., 2015; Lozano et al., 2019; Ling et al., 2020; Wang et al., 2020). For rodents with chronically elevated intraocular pressure, morphological changes were identified in LC tissues (Dai et al., 2012; Pang and Clark, 2020; Zhang et al., 2022). Changes in the microstructure of tissues will affect their mechanical properties. Liu et al. found that the nonlinear mechanical behavior of the cornea is closely related to the curled morphology of collagen fibrils (Liu et al., 2014). Therefore, mechanical properties of LC may change with the duration of elevated IOP.

It has been found that the Young’s modulus of LC in patients with pseudoexfoliation glaucoma decreases by 40% compared to normal eyes (Braunsmann et al., 2012). Jia et al. found that mechanical properties of peripapillary sclera and LC in monkeys varied with high IOP lasting 40 days (Jia et al., 2022). Recent research also found strains of mouse astrocytic LC in an *ex vivo* inflation test was greater than the control in the central astrocytic LC after 3 days of *in vivo* elevated IOP (Korneva et al., 2023). However, changes in mechanical properties of the LC and RGC axons with the duration of elevated IOP remain unclear.

To study changes in mechanical properties of the LC and RGC axons with the duration of elevated IOP, an animal model with chronic high IOP was established. The rat chronic high IOP animal model could be induced by injection of microbeads, laser photocoagulation, or episcleral vein cauterization (Ueda et al., 1998; Blanco et al., 2019; Pang and Clark, 2020; Rodrigo et al., 2021; Korneva et al., 2023). Our previous studies have induced the model by cauterizing episcleral veins with 5-Fluorouracil (5-Fu) subconjunctival injection, which may elevate the episcleral venous pressure and then obstruct the outflow of aqueous humor (Li et al., 2018; Peng et al., 2019; Ma et al., 2025). This model is reproducible and the high IOP of the model could be sustained for 3 months (Zhang et al., 2022). We called the rat LC tissue “glial LC” because the fortified astrocytes are the main component of the LC tissue (Liu et al., 2022). Additionally, we should verify whether 5-Fu affects the morphology and mechanical properties of the LC tissue in this paper.

It is difficult to measure the mechanical properties of LC using traditional mechanical methods because it is a multi-layered network structure with many pores (Quigley et al., 1981; Downs and Girkin, 2017). Braunsmann et al. measured the Young’s modulus of the LC beams of normal human eyes and pseudo-exfoliated eyes by atomic force microscope (AFM), which is an effective tool for investigating the mechanical properties of small biological samples (Ziebarth et al., 2007; Last et al., 2009, 2011; Grant et al., 2011; Braunsmann et al., 2012). To analyze the influence of high IOP on the LC deformation more accurately and improve the prediction performance for optic nerve damage, we have proposed a method combining AFM with the Otsu image segmentation method to distinguish mechanical properties of glial LC and RGC axons in the glial LC area of rats (Liu et al., 2022).

This study established a chronic high IOP animal model by cauterizing episcleral veins with 5-FU subconjunctival injection and studied the effect of 5-FU on the morphology and mechanical properties of the LC tissue. Then we quantitatively evaluated the mechanical properties of glial LC and RGC axons, at different times with duration of high IOP, which might provide an important basis for in-depth study of the pathogenesis of optic nerve damage and early diagnosis of glaucoma.

## 2 Materials and methods

### 2.1 Animals

Male adult Sprague-Dawley rats (7–8 weeks, 270–300 g weight) were obtained from the Experimental Animal Department of the Capital Medical University. They were housed libitum and maintained in an air-conditioned room in a 12-h light/12-h dark cycle. Animal experiments conformed to the principles of animal treatment described in the Statement for Use of Animals in Ophthalmic and Vision Research of the Association for Research in Vision and Ophthalmology.

A total of 67 rats were involved in this experiment. According to different duration of high IOP, they were divided into four groups, including a blank control group, 4th, 8th, and 12th week after high IOP induction, denoted by Gw0, Gw4, Gw8, and Gw12, respectively. The right eye served as the experimental eye, while the left eye functioned as the contralateral control eye in the Gw4, Gw8, and Gw12 groups. To verify the feasibility of the model induction related to the injury of the posterior tissue of the eyeball, we established a 5-FU drug control group, named Gw0-5Fu. Table 1 shows the number of rats in each group prepared for hematoxylin-eosin (HE) staining, immunofluorescence staining, transmission electron microscopy testing, and AFM testing.

**TABLE 1:** The number of rats in each group rat prepared for HE staining, immunofluorescence staining, transmission electron microscopy testing, and AFM testing.

Group	Gw0	Gw4		Gw8		Gw12		Gw0-5Fu
	Right	Right	Left	Right	Left	Right	Left	Right
HE staining	3	3	0	3	0	3	0	3
Immunofluorescence staining	3	3	0	3	0	3	0	3
Transmission electron microscopy testing	1	0	0	0	0	1	0	1
AFM testing	10	6	6	6	6	6	6	6

Note: Gw0 denotes blank control group; Gw4 denotes the experimental group with high IOP, for 4 weeks; Gw8 denotes the experimental group with high IOP, for 8 weeks; Gw12 denotes the experimental group with high IOP, for 12 weeks; GW0-5Fu denotes 5-FU, drug control group, which means the right eyes of rats were injected 5-Fu alone. Gw0, Gw4, Gw8, Gw12, and Gw0-5Fu used a total of 17, 12, 12, 13, and 13 rats, respectively.

### 2.2 Model induction and IOP measurements

The rat model with chronic high IOP was established by cauterizing the episcleral venous in combination with subconjunctival injection of 5-Fu (Haipu Pharmaceutical Co., Shanghai, China), which was described in detail in previous studies (Li et al., 2018; Peng et al., 2019; Liu et al., 2022). Rats were anesthetized intraperitoneally with 1% sodium pentobarbital at a dose of 0.4 ml per 100 g weight. The surface anesthesia was performed on the cornea and periocular tissues by adding oxybuprocaine hydrochloride eye drops (Santen Pharmaceutical, Osaka, Japan) to the experimental eye. The experimental eye was cauterized with a high-temperature electrocoagulation pen on 3-4 upper scleral vein trunks. A 29G needle was used to administer a 100 μL subconjunctival injection of 5-Fu at a concentration of 2.5% (w/v) into the eyes to suppress neovascularization. Then a subconjunctival injection of 100 μL of 5-Fu with a concentration of 2.5% (w/v) was performed by a 29G needle on the eyes to inhibit neovascularization. Levofloxacin eye drops were added to the ocular surface to prevent inflammation.

IOP was measured using a TonoLab Rebound Tonometer (Icare, Vantaa, Finland) every 3 days after induction. To avoid the effects of circadian rhythms, IOP measurements in awake rats were scheduled between 10 am and 12 am. If IOP was lower than 30 mmHg, cauterization was performed again. Otherwise, only 5-Fu was injected. For the 5-Fu drug control group, Gw0-5Fu, only 5-Fu was injected subconjunctivally to prevent the formation of new blood vessels.

### 2.3 HE staining

Histological changes in the ONH were assessed through HE staining in conjunction with optical microscopy. Three rats were selected randomly from every group to obtain coronal and sagittal sections of the ONH. Following ONH fixation, dehydration was carried out progressively using different ethanol concentrations (Macklin, Shanghai, China), followed by transparency using xylene (Solarbio, Beijing, China). After thorough infiltration with wax, the tissue was embedded. The ONH was sliced into 4 μm-thick sections using a paraffin slicing machine (Leica, Wetzlar, Germany). Paraffin sections were rinsed three times in PBS and then incubated with 3% hydrogen peroxide. Subsequently, the sections were immersed in hematoxylin (Push, Chengdu, China) and differentiated in 1% hydrochloric acid alcohol. After staining with eosin (Push, Chengdu, China), the slides were dehydrated using progressively concentrated ethanol and clarified with xylene. Neutral resin adhesive was employed to seal the slides.

### 2.4 Immunofluorescence staining

The tissues in the LC region of the rat are primarily composed of astrocytes and the RGC axons. To investigate morphology changes in the ONH, immunofluorescence staining of glial fibrillary acidic protein (GFAP) was performed on coronal plane sections of the ONH. A series of 10 μm cryo-sections were sliced. These cryo-sections were rinsed three times in PBS, sealed in 5% bovine serum albumin (BSA; Sigma, Saint Louis, USA) in PBS with 0.3% Triton X-100, and transferred to a primary antibody solution consisting of rat anti-glial fibrillary acidic protein (Abcam, Cambridge, UK) diluted with PBS (1:500). The sections were then incubated with the primary antibodies overnight at 4°C in a humidified box. Following the primary incubation, the sections were rinsed and incubated with species-specific secondary antibodies conjugated with Alexa-488 (1:500) (Abcam, Cambridge, UK) at 37°C for 2 h. Fluorescent images of the labeled tissues were visualized and captured using confocal laser scanning microscopy (CLSM; Leica, Wetzlar, Germany). Images of the eyes in every group were acquired under the same settings and conditions.

### 2.5 Transmission electron microscopy

To visualize the microstructure of the ONH, we used a transmission electron microscope (TEM, HT7700, HITACHI, Japan) to observe images of the ONH using three rats selected from the Gw0 group, the Gw12 group, and the Gw0-5Fu group, respectively. The tissue was prepared following TEM specifications, where it was fixed in 2.5% glutaraldehyde for 4–6 h and washed three times with PBS. Subsequently, the ONHs were fixed with 1% osmium acid. Following elution with gradient ethanol, the ONH was immersed in acetone, embedded, and solidified using Epon618 (Sigma, Saint Louis, USA). After determining the position, the tissue was sliced into approximately 50 nm-thick ultra-thin sections. These ultra-thin sections were then stained with lead citrate.

### 2.6 Mechanical properties measurement of glial LC and RGC axons

The mechanical properties of glial LC and RGC axons were obtained using an Atomic Force Microscope (AFM, Bioscope Resolve, Bruker, America) and image processing method based on our previous research (Liu et al., 2022). We chose a quadrangular pyramid probe (MLCT-A, Bruker, America) with a soft silicon nitride triangular cantilever whose spring constant is 0.07 N/m. The indentation velocity was set as 29.6 μm/s. We selected a soft silicon nitride triangular cantilever with a spring constant of 0.07 N/m and a four-sided pyramidal probe with a height of 2.5–8.0 μm (MLCT-A, Bruker, USA). It is worth noting that while the probe is tapered, its tip has a curvature radius of 20 nm to prevent stress concentration. The predefined maximum force for each indentation (trigger force) was set at 2.5 nN, and the indentation speed was 29.6 μm/s. In the force volume (FV) mode of AFM, the probe on the tip of the cantilever performed a two-dimensional scan of the sample within a region of interest (ROI) and acquired indentation curves (F-δ curve). Then, a Young’s modulus image, each pixel value representing a local Young’s modulus, is obtained by fitting the corresponding F-δ curve. In this experiment, an ROI with the size of 20 × 20 μm^2^ was selected and indentations were performed on 128 × 128 points. The Young’s modulus of glial LC and RGC axons were segmented using the Otsu thresholding segmentation method based on the Young’s modulus image. The mechanical properties of glial LC and RGC axons were determined according to the respective Young’s modulus extracted using statistical analysis.

### 2.7 Statistical analyses

A one-way analysis of variance (ANOVA) was conducted to compare data across multiple groups, with statistical significance set at P < 0.05. Post hoc pairwise comparisons were performed using the least significant difference (LSD) test, with a threshold of P < 0.05 indicating statistical significance.The correlation between the mechanical properties of glial LC or RGC axons and the duration time of high IOP was tested by using Spearman correlation with SPSS 26.0 (IBM Corporation, Armonk, United States).

## 3 Results

**FIGURE 1:**
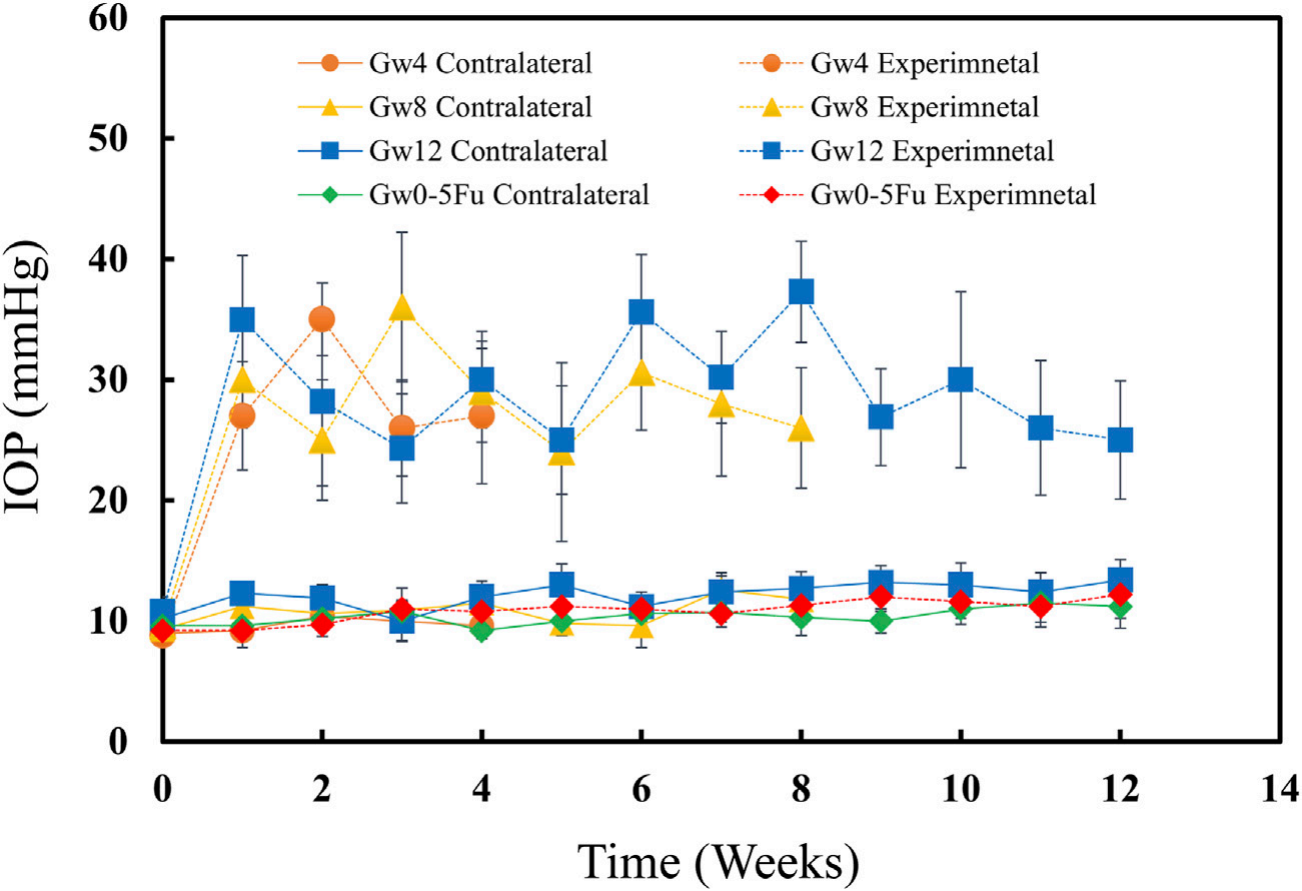
IOPs of both eyes in the 5-Fu group and experimental groups (n = 13). The IOPs of experimental eyes increased significantly after model induction (P < 0.05) and the IOPs of contralateral control eyes remained at normal level (P > 0.05). The IOPs of 5-Fu eyes showed no significant change after model induction (P > 0.05). Error bars represent the standard deviation (SD).

IOPs of eyes were measured when the rats were awake before the experiment. IOPs had no significant change between Gw0-5Fu contralateral eyes and Gw0-5Fu experimental eyes until 12 weeks, indicating that injection of 5-Fu did not affect IOP (Figure 1). IOPs of experimental eyes increased after inducing chronic high IOP model and sustained high IOP level for 12w. The contralateral eyes maintained normal IOP, indicating that the method of inducing the model did not affect the IOP level of the contralateral eyes in this study (Figure 1).

**FIGURE 2:**
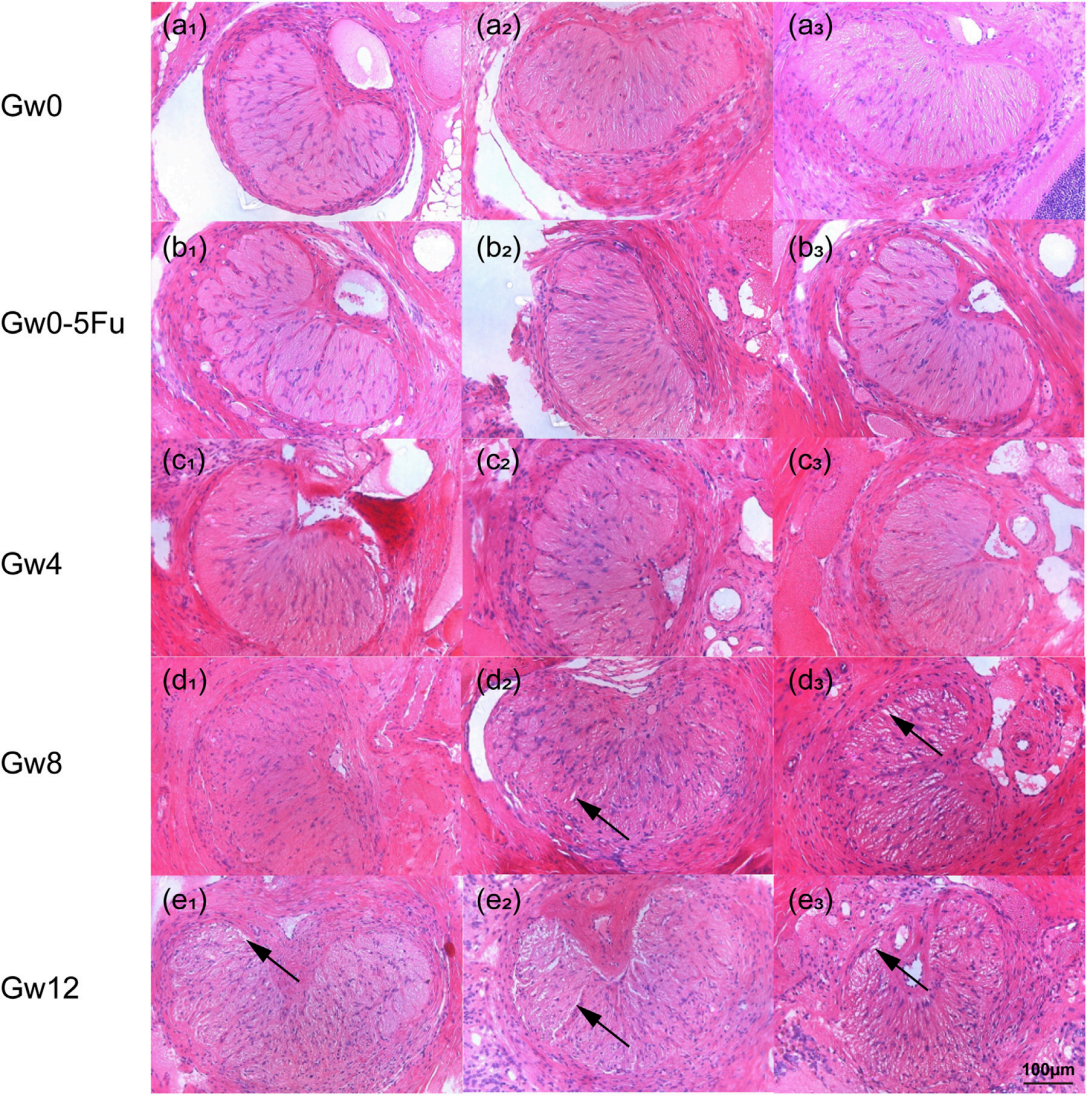
The HE staining images of cross sections in the LC tissues in all groups (n = 3). **(a1-3)** represents the Gw0 group, **(b1-3)** is the Gw0-5Fu group, while **(c1-3)**, **(d1-3)**, and **(e1-3)** correspond to the high IOP experimental groups at 4 weeks, 8 weeks, and 12 weeks, respectively. In the figure, the blue-purple color represents cell nuclei, the pink region is mainly composed of the elongated axons of astrocytes, and the white arrows indicate the gaps appearing in the LC.

By following similar procedures, the results of HE staining presented a kidney-like shape in the cross-sectional view of the rat LC (Figure 2), which consists of glial LC and RGC axons (Dai et al., 2012; Peng et al., 2019). The circular or elliptical structures in blue-purple represent cell nuclei, and the cell types mainly included astrocytes, a small number of small glial cells, and vascular endothelial cells (Williams et al., 2017; Lopez et al., 2020; Strickland et al., 2022). The pink region mainly consists of elongated astrocytic protrusions. The morphology of the LC tissue in the Gw0-5Fu group exhibited no significant differences compared to the Gw0 group. Similarly, regular radial glial fiber processes were observed in the Gw0-5Fu group, along with a similar distribution of cell nuclei. The morphological structure of LC tissues has changed significantly with the duration of high IOP. Apart from the central region of LC tissue, the peripheral areas present a large number of cell nuclei, indicating the activation of cell proliferation and the increase of cell population. A large number of white voids appeared in the LC area, indicating that the structure of LC tissue had undergone remodeling, and the RGC axons might also be greatly affected (Figure 2).

**FIGURE 3:**
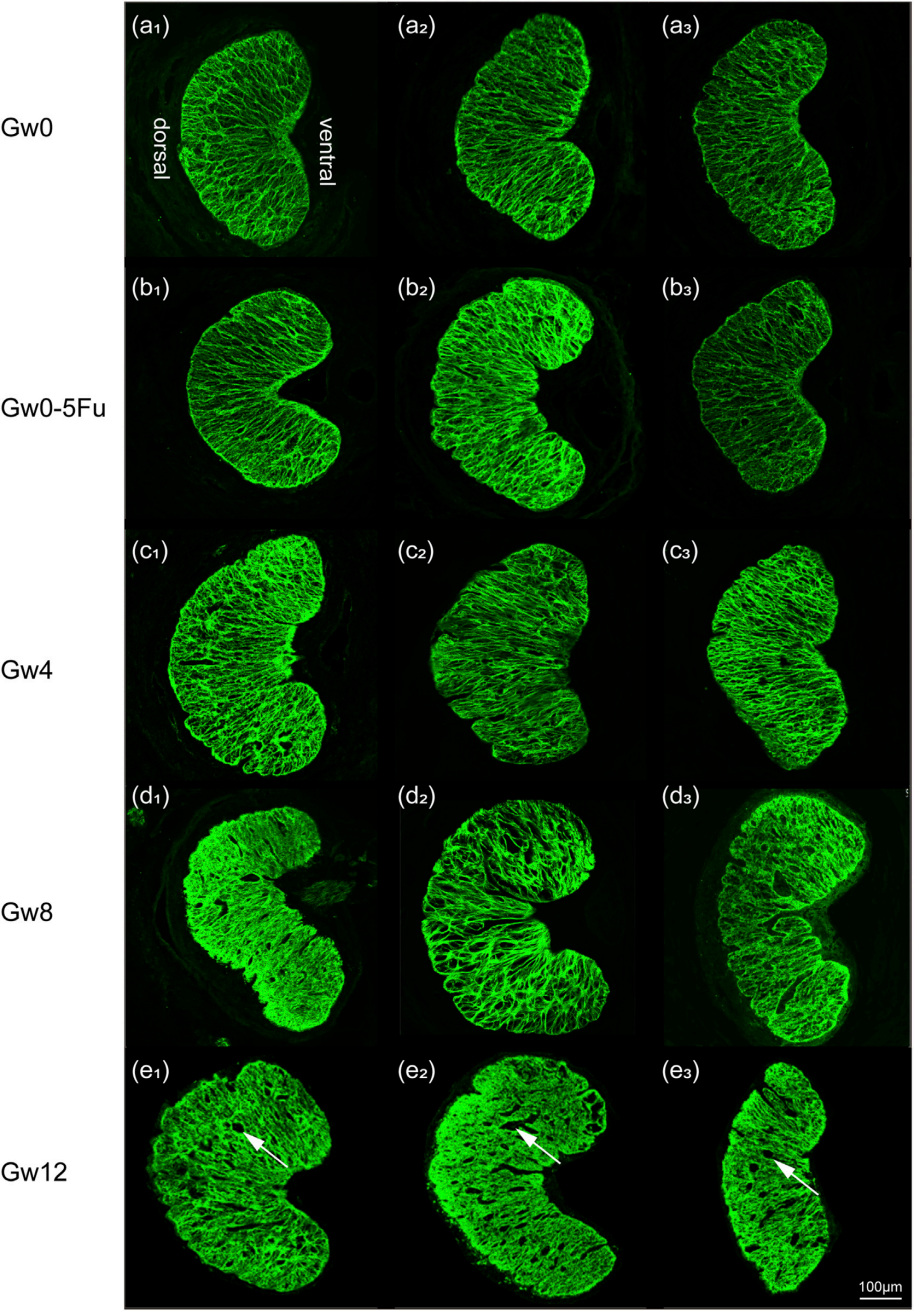
The fluorescent images of cross sections in the LC tissues in all groups (n = 3). Overall images in all groups show the variation of meshwork of astrocytes (green). Images **(a1-3)** represent the Gw0 group, **(b1-3)** is the Gw0-5Fu group, while **(c1-3)**, **(d1-3)**, and **(e1-3)** correspond to the high IOP experimental groups at 4 weeks, 8 weeks, and 12 weeks, respectively. In the figure, white arrows represent the voids and gaps that appeared in the LC.

To observe morphological changes in the glial LC intuitively, we applied immunofluorescent staining to label the GFAP protein of glial LC and observe the morphological changes through a laser confocal microscope. There were no significant differences in the distribution and arrangement of the glial LC beam between the Gw0-5Fu group and the Gw0 group. The astrocyte protrusions extended radially from the ventral side to the dorsal side, forming a reticular pattern with a relatively uniform and distinct hierarchical structure. With the duration of high IOP, the radial arrangement structure was destroyed and the staining was blurred. While high IOP lasted for 12 weeks, voids and gaps appeared after a large number of fibers were lost, and the original support structure of the glial LC was destroyed (Figure 3).

**FIGURE 4:**
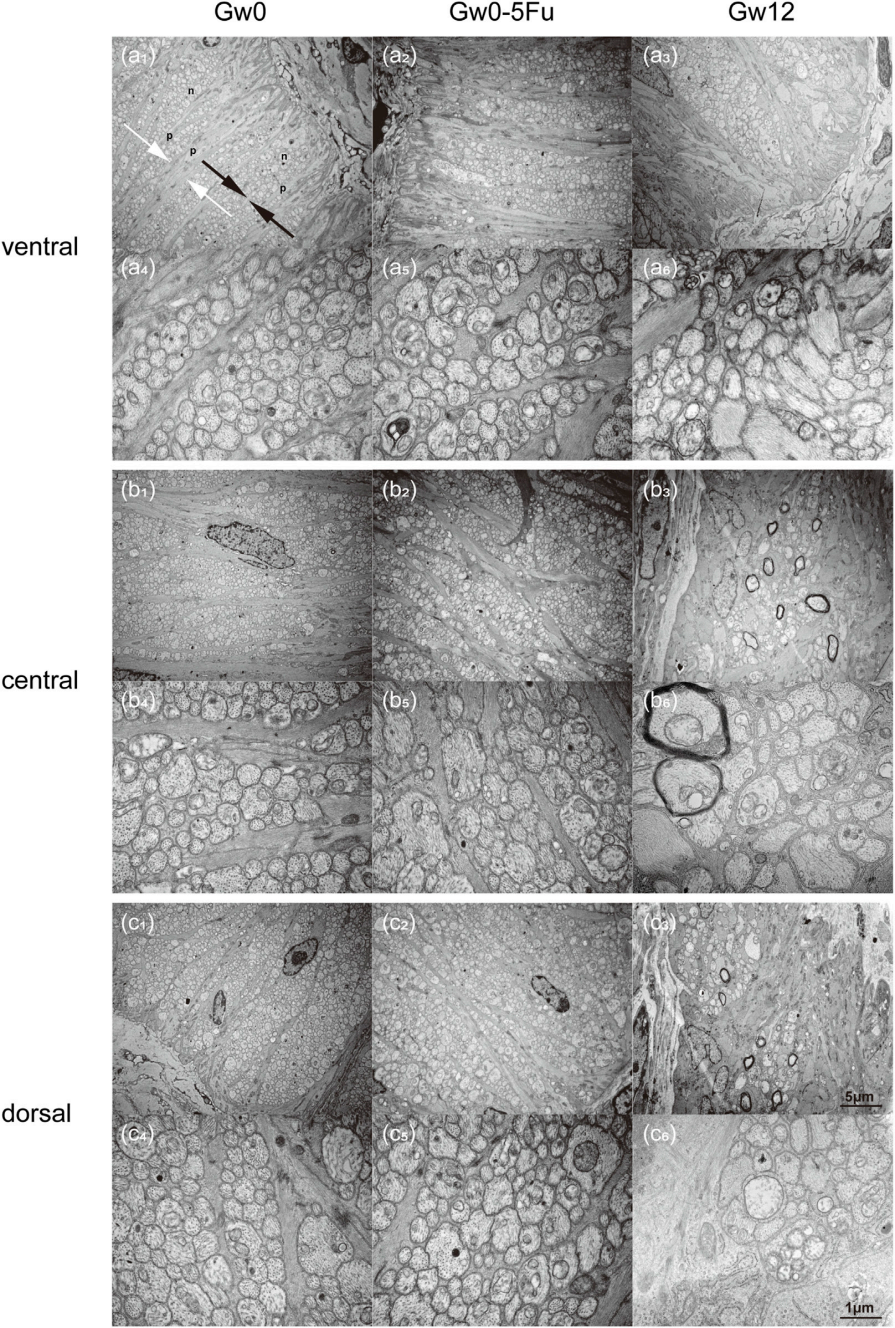
Transmission electron microscopy images of the glial LC in the experimental eyes in the Gw0, Gw0-5Fu, and Gw12 groups. Images **(a1-6)**, **(b1-6)**, and **(c1-6)** depict the ventral, central, and dorsal regions, respectively. Images **(a1-3)**, **(b1-3)**, and **(c1-3)** are magnified at 6,000 times, while images **(a4-6)**, **(b4-6)**, and **(c4-6)** show local regions at a magnification of 30,000 times. In the figure, ‘p’ represents the protrusions of astrocytes; ‘n’ represents RGC axons; white arrows represent bundles composed of protrusions of astrocytes; black arrows represent bundles of RGC axons.

To further insight into the microstructural changes of RGC axons and astrocytes, we conducted more detailed observations using TEM on the cross-section of the ventral, central, and dorsal areas of the LC tissues in the Gw0, Gw0-5Fu, and Gw12 groups. The ventral area of the LC tissues in the Gw0 group revealed a dense and sturdy “root” of astrocytes, extending dorsally with the organized arrangement. The protrusions of astrocytes were relatively robust (marked as “p” in Figures 4a1,a4), with a single bundle diameter of 0.3–0.6 μm. The diameter of bundled protrusions (indicated by white arrows in Figures 4a1,a4) was 3–5 μm. Additionally, there were fine cell protrusions closely surrounding the bundles of RGC axons (indicated by black arrows in Figures 4a1,a4). Numerous RGC axons (marked as “n” in Figures 4a1,a4) shuttled in a circular bundle within the spindle-shaped gaps of astrocytic protrusions. Most RGC axons passed perpendicularly through the glial LC, whose diameter varied from 0.2 μm to 2 μm in cross-section. In the central area of glial LC, astrocytic protrusions continuously dispersed into finer bundles as they radiate from the ventral to the dorsal area, resembling branching tree structures. In the dorsal area of glial LC, astrocytic protrusions were finer and more sparsely distributed compared to the ventral area (Figures 4b1,b4). In contrast, RGC axons were denser in the dorsal area compared to the ventral area (Figures 4c1,4c4). The distribution of astrocytic protrusions in the Gw0-5Fu group resembled that of the Gw0 group. The outer sheath structure of RGC axons in the Gw0-5Fu group remained intact, with no loss of axons, and its distribution density was similar to that of the Gw0 group. It was indicated that 5-Fu did not cause significant changes in the morphology of RGC axons (Figures 4a2,a5,b2,b5,c2,c5). Compared to the Gw0 group, the Gw12 group exhibited a disrupted radial structure of astrocytic protrusions in the central and dorsal areas (Figures 4a3,b3,c3). The results show that both thickness and density of astrocytic protrusions are increased, which could compress and encroach upon the space of RGC axons. The sectional shape of part of RGC axons varied from ellipse to irregular shapes (Figures 4a6,b6,c6).

**FIGURE 5:**
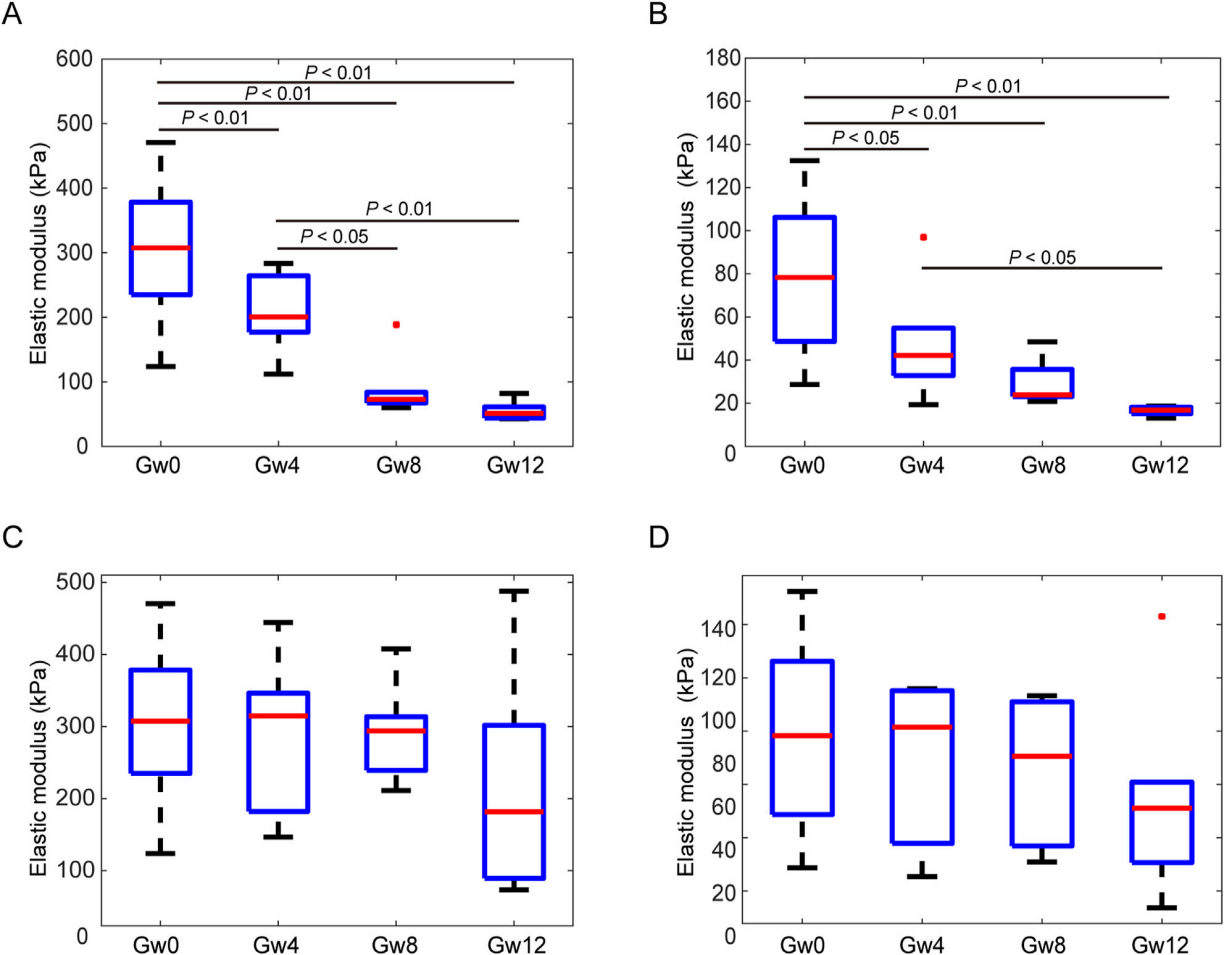
Comparison of the Young’s modulus between experimental eyes at 4 weeks, 8 weeks, and 12 weeks and contralateral control eyes in the glial LC and RGC axons. **(A)**. The Young’s modulus of the glial LC in the experimental eyes in the Gw0, Gw4, Gw8, and Gw12 groups. **(B)**. The Young’s modulus of the RGC axons in the experimental eyes in the Gw0, Gw4, Gw8, and Gw12 groups. **(C)**. The Young’s modulus of the glial LC in the contralateral control eyes in Gw0, Gw4, Gw8, and Gw12 groups. **(D)**. The Young’s modulus of the RGC axons in the contralateral control eyes in Gw0, Gw4, Gw8, and Gw12 groups.

Next, we obtained the changes of Young’s modulus of glial LC and RGC axons with the duration of high IOP (Figures 5A,B). The values of Young’s modulus were taken as the typical Young’s modulus, which is the peak of the fitted frequency distribution histogram of Young’s modulus (Liu et al., 2022). With the sustained elevation of IOP, the Young’s modulus of the experimental group of both glial LC and RGC axons significantly decreased (Figures 5A,B). The Young’s modulus of glial LC are greater than those of RGC axons. The difference in Young’s modulus of glial LC between the experimental group and control group is statistically significant (P′ < 0.01). The variation of Young’s modulus of glial LC with duration time of high IOP was similar to that of glial LC. There was no significant difference in the Young’s modulus between the Gw8 group and the Gw12 group whether it was for the glial LC or RGC axons.

**FIGURE 6:**
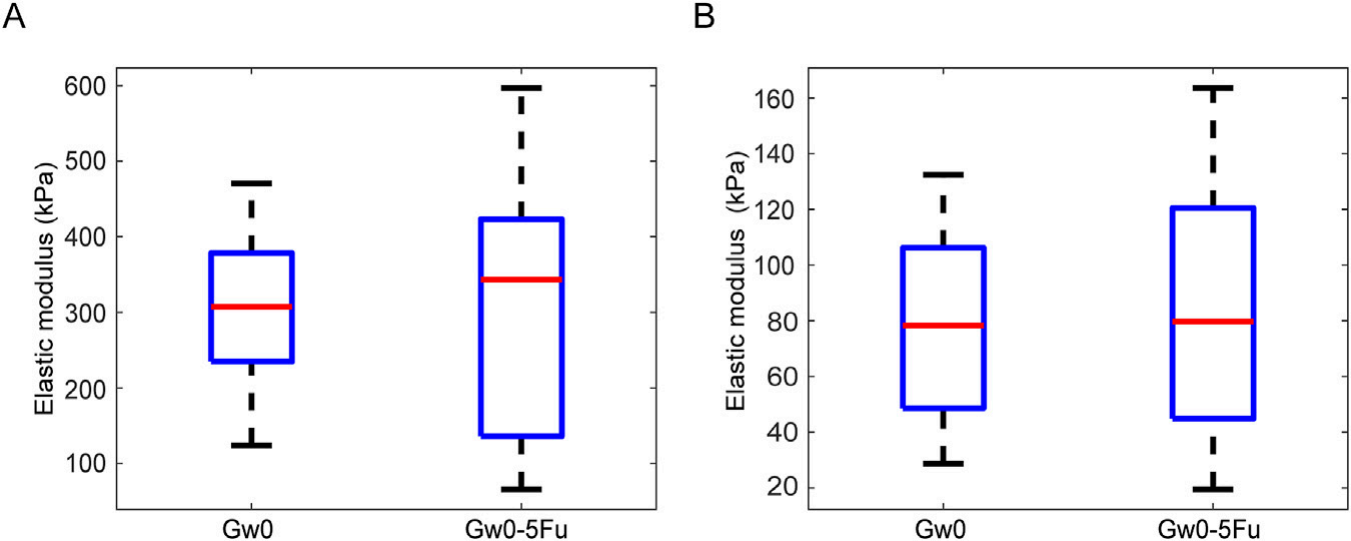
Comparison of the Young’s modulus between the blank control group and 5-FU control group in the glial LC and RGC axons. **(A)**. The Young’s modulus of the glial LC in the experimental eyes in the Gw0 and Gw0-5Fu groups. **(B)**. The Young’s modulus of the RGC axons in the contralateral control eyes in the Gw0 and Gw0-5Fu groups.

The Young’s modulus showed no significant differences for different duration of IOP in either the glial LC or RGC axons in the contralateral eyes (Figures 5C,D). Additionally, both the glial LC and RGC axons showed no significant differences in Young’s modulus between the Gw0 group and the Gw0-5Fu group, indicating that 5-FU had no obvious influence on the mechanical of the glial LC and RGC axons (Figures 6A,B).

The correlations between the Young’s modulus of the tissues and the duration time of high IOP were further analyzed by Spearman correlation analysis for the experimental group and the control group. The results are shown in Table 2. The Young’s modulus of the glial LC and RGC axons in the experimental group are negatively correlated with the duration time of high IOP, and the correlation is statistically significant (P < 0.01). There is no correlation between the Young’s modulus of glial LC and RGC axons in the contralateral eye and the duration of high IOP.

**TABLE 2:** Correlation analysis of Young’s modulus of glial LC and RGC axon with duration time of high IOP.

Group	r	P
Glial LC of experimental eyes	−0.795	**
RGC axon of experimental eyes	−0.773	**
Glial LC of contralateral eyes	−0.226	
RGC axon of contralateral eyes	−0.239	

*Note the Spearman correlation coefficients had statistically significant (*P < 0.05, **P < 0.01).

## 4 Discussion

In this study, a rat model with chronic high IOP was established by cauterizing the superior scleral vein combined with subconjunctival injection of 5-FU, and the reliability of the modeling method was verified by combining HE staining, immunofluorescence staining, and TEM. With the duration of high IOP, the morphological structure of LC tissues has undergone significant changes. We used AFM combined with image segmentation methods to study the linear elastic mechanical properties of the glial LC and RGC axons in normal eyes and experimental eyes with different durations of high IOP. The results showed that the Young’s modulus of glial LC and RGC axons decreased significantly with the duration of high IOP.

The IOP of the experimental eyes in the Gw0-5Fu group was not significantly different from that in the Gw0 group. Following the injection of the 5-Fu drug, there was no observable bleeding or edema around the bulbi conjunctiva, similar to normal eyes. Results from HE staining and GFAP immunofluorescence staining experiments indicate that the LC tissues in the Gw0-5Fu group are not different from those in the Gw0 group. Results of TEM reveal that in different regions of the LC tissue, there was no apparent loss of optic nerve fibers in the RGC axon bundles in the Gw0-5Fu group. Based on these findings, it can be inferred that 5-FU does not cause significant damage to RGC axons. Studies have shown that the efficacy of 5-FU occurs only when it is converted into active nucleoside metabolites in various tumor lesions (Sethy and Kundu, 2021; Alzahrani et al., 2023). If there are no lesions, they will quickly penetrate the blood-brain barrier and enter the brain tissue after being injected into the body, followed by 10%–30% of the prototype drug being excreted in the urine through the kidneys, and about 60%–80% was inactivated in the liver and decomposed into carbon dioxide and urea, which are excreted through the respiratory tract and urethra respectively (Matuo et al., 2009; Sethy and Kundu, 2021). 5-FU is a chemotherapeutic agent primarily employed in the treatment of various cancers, including colorectal, gastric, and breast cancers (Sommadossi et al., 1982; Petrelli et al., 1987; Kim et al., 1993; Prado et al., 2007). As a cell cycle-specific agent, 5-Fu primarily targets cells with high mitotic activity, like retinal epithelial cells. Astrocytes, which make up the rat glial LC, do not undergo proliferation during the development of glaucoma (Inman and Horner, 2007). Therefore, 5-Fu has no effect on cell proliferation within the LC tissue. Furthermore, it takes an injection of 60 mg/kg to trigger a noticeable inflammatory reaction in rats (Kırcadere et al., 2024). Since we injected only 2.5 mg of 5-Fu per mouse, we believe that the 5-Fu used in this study is unlikely to cause inflammation in the glial LC and RGC axons. In order to study the 5-Fu impact on the results, we design the 5-Fu control group.

A method for estimating mechanical properties of ONH tissues from parameters measurable using optical coherence tomography showed the average Young’s modulus was 0.24 MPa for human LC (Sigal et al., 2014). The results showed that the Young’s modulus of the LC tissues decreased with the duration of chronic elevated IOP, which was consistent with the Young’s modulus of the LC tissues observed in patients with pseudoexfoliation glaucoma clinically (Braunsmann et al., 2012). The changes in mechanical properties of the LC tissues may be related to morphological alterations. The pore area fraction of the lamina cribrosa (LC) was significantly correlated with pressure-induced strains. Specifically, all strain measures increased with higher pore area fraction. This suggests that regions with a larger pore area fraction, indicating a less dense collagen network, are associated with greater susceptibility to deformation under elevated intraocular pressure, potentially contributing to glaucomatous damage (Ling et al., 2019). Our study revealed that with the duration of chronic elevated IOP, there is an increase in the expression of GFAP, disruption of the radial arrangement structure, and severe loss of RGC axons. The previous research also suggested that the glial LC was damaged with the duration of chronic elevated IOP (Guan et al., 2022; Zhang et al., 2022). Several researches indicated a correlation between changes in microstructure and mechanical properties. The nonlinear mechanical behavior of the cornea was closely correlated with the crimping morphology of collagen fibrils (Liu et al., 2014). Studies have shown that changes in the mechanical properties of the sclera in myopic eyes are closely related to alterations in its microstructure (Boote et al., 2020; Hoerig et al., 2022). Moreover, studies have shown that elevated IOP is significantly associated with ECM changes in the ONH. In the glaucomatous rat model with elevated IOP, deposition of TGF-β2 and collagen I in the ONH was significantly increased (P < 0.01), while collagen IV and MMP-1 showed no significant changes (Guo et al., 2005; Kim and Lim, 2022). These findings suggest that IOP-induced ECM remodeling may play an important role in ONH damage in glaucoma. Therefore, the underlying mechanisms of changes in the mechanical properties of LC and RGC axons, particularly extracellular matrix remodeling or alterations in the cytoskeleton, require further exploration.

Similar to human LC tissues, the structures of rat LC tissues exhibit significant differences in both cross-sections and longitudinal cross-sections (Morrison et al., 1995; Guan et al., 2022; Czerpak et al., 2023). Therefore, we investigated the anisotropy of the mechanical properties of rat LC tissues. Statistical analysis results indicate that there are no significant differences in Young’s modulus in longitudinal cross-sections and cross-sections for glial LC or RGC axons (Supplementary Figure 1). The possible reason could be that the contact radius of indentation is 20–150 nm, which is related to the half of angle of the quadrangular pyramid probe depth (about 17°) depth (Liu et al., 2022). The maximum diameter of astrocyte protrusions (glial LC) could reach 0.3–0.6 μm, and the maximum short axis of the RGC axon bundle could reach 0.2–2 μm. Therefore, we believe that each indentation of the probe used in AFM measurement might press on the single astrocyte protrusions or RGC axon bundle of the glial LC or RGC axon. Therefore, there is no significant difference between the mechanical properties of tissues obtained from the longitudinal section or cross-section.

There are some differences between the induced elevated IOP in this study and the naturally occurring elevated IOP in glaucoma. Induced elevated IOP is usually limited to ocular pathological research and cannot reflect the complex effects of systemic metabolic abnormalities or the neuroendocrine axis on IOP regulation (McDermott et al., 2024). In contrast, elevated IOP in glaucoma is significantly associated with systemic diseases such as hypertension and diabetes (Umetsu et al., 2024). Additionally, compared to the induced elevated IOP, the elevated IOP in glaucoma lasts longer and is influenced by age-related factors. Moreover, there are also some differences between the LC in humans and rats. In primates, the LC primarily consists of collagen, with a dense population of astrocytes on its surface and LC cells within the LC (Hernandez et al., 2008). In contrast, the equivalent structure in rodents, known as the glial LC, is composed almost entirely of astrocytes and lacks collagen (Howell et al., 2007). We found that the mechanical properties of LC tissues in rats decreased with the duration of high IOP, which was consistent with the results of Braunsmann et al. (Braunsmann et al., 2012). Their results indicated that the Young’s modulus of LC tissues in Pseudoexfoliation (PEX) eyes had a significant decrease compared with normal eyes.

There are some limitations in our study. First, the minimum duration of chronic elevated IOP in rats is 4 weeks, making it insufficient to observe alterations in morphology and mechanical properties of LC tissues within a shorter time. Second, we investigated the linear mechanical properties of LC tissues under chronic elevated IOP due to small indentation. Nonlinear and viscoelastic mechanical properties need to be further investigated with bigger indentation depth or other methods.

In summary, we found that mechanical properties of LC tissues in rats have changed significantly under long-term effects of high IOP. Compared with the control group, the Young’s modulus of the glial LC decreased by 35.5%, 74.2%, and 80.6% after 4, 8, and 12 weeks of elevated IOP, respectively; the Young’s modulus of RGC axons decreased by 45.6%, 70.9%, and 75.9% after 4, 8, and 12 weeks of elevated IOP, respectively. In addition to the supporting structures, RGC axons may also have been severely damaged. In the future study, we will set up more intensive time points of high IOP to further study the physiological changes of astrocytes and RGC axons under the effect of high IOP to provide the basis for further understanding the mechanism of the injury of the glaucoma optic nerve.

## Data Availability

The original contributions presented in the study are included in the article/Supplementary Material, further inquiries can be directed to the corresponding author/s.
