# Supplementary material for: Mechanical property changes of glial LC and RGC axons in response to high intraocular pressure
Source: Front Bioeng Biotechnol. 2025 Apr 28;13:1574231. doi: 10.3389/fbioe.2025.1574231 (PMC12066477; doi:10.3389/fbioe.2025.1574231)
Supplement: Supplementary file 1 [file DataSheet1.docx]

Supplementary Material


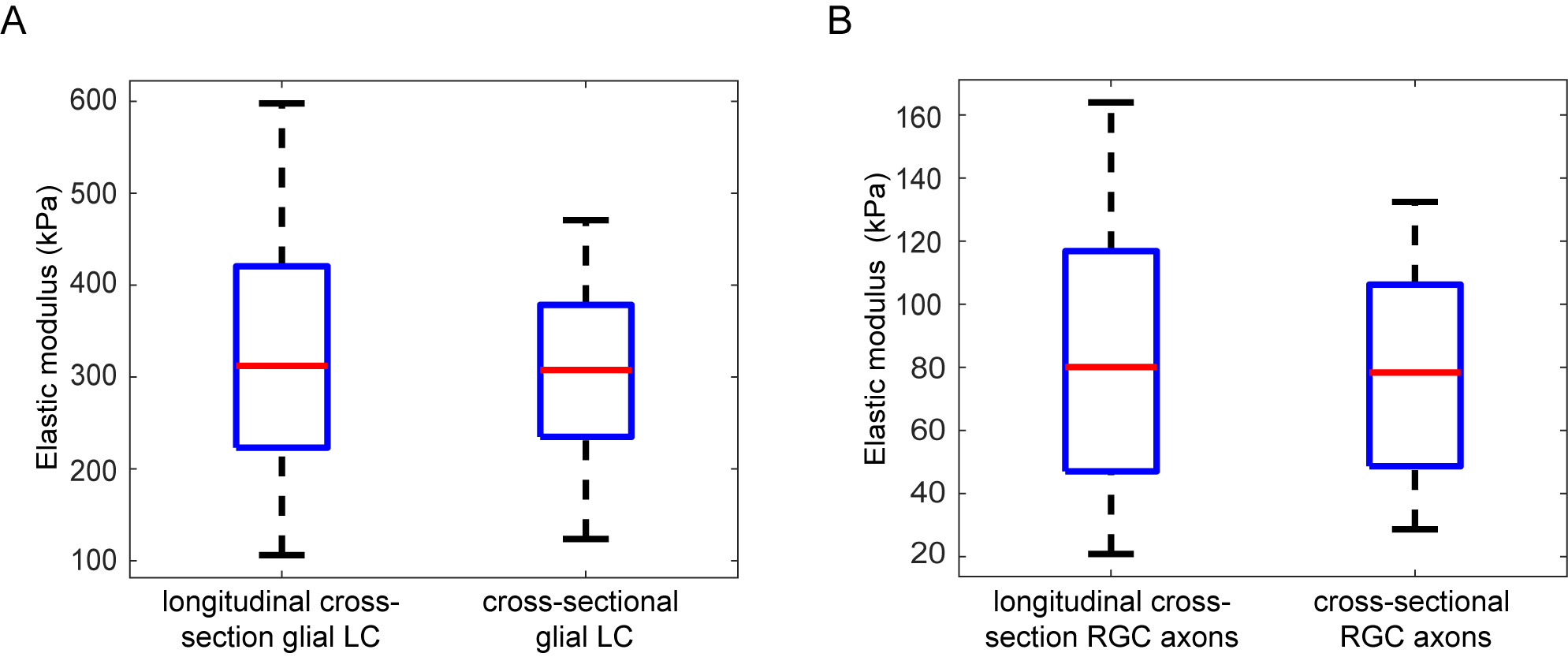


**Supplementary Figure 1.** The Young’s modulus of cross-sections and longitudinal cross-sections of the glial LC and RGC axons.
